# Liver segmentation: indications, techniques and future directions

**DOI:** 10.1007/s13244-017-0558-1

**Published:** 2017-06-14

**Authors:** Akshat Gotra, Lojan Sivakumaran, Gabriel Chartrand, Kim-Nhien Vu, Franck Vandenbroucke-Menu, Claude Kauffmann, Samuel Kadoury, Benoît Gallix, Jacques A. de Guise, An Tang

**Affiliations:** 1 Department of Radiology, Radio-oncology and Nuclear Medicine, University of Montreal, Saint-Luc Hospital, 1058 rue Saint-Denis, Montreal, QC H2X 3J4 Canada; 2 Department of Radiology, McGill University, Montreal General Hospital, 1650 Cedar Avenue, Montreal, QC H3G 1A4 Canada; 3 University of Montreal, 2900 boulevard Eduoard-Montpetit, Montreal, QC H3T 1J4 Canada; 4 Centre de recherche du Centre Hospitalier de l’Université de Montréal (CRCHUM), 900 rue Saint-Denis, Montreal, QC H2X 0A9 Canada; 5 Imaging and Orthopaedics Research Laboratory (LIO), École de technologie supérieure, Centre de recherche du Centre Hospitalier de l’Université de Montréal (CRCHUM), 900 rue Saint-Denis, Montreal, QC H2X 0A9 Canada; 6 Department of Hepato-biliary and Pancreatic Surgery, University of Montreal, Saint-Luc Hospital, 1058 rue Saint-Denis, Montreal, QC H2X 3J4 Canada; 7 École Polytechnique de Montréal, University of Montreal, 2500 chemin de Polytechnique Montréal, Montreal, QC H3T 1J4 Canada

**Keywords:** Liver, Segmentation, Volumetry, Automated, Computed tomography, Magnetic resonance imaging

## Abstract

**Objectives:**

Liver volumetry has emerged as an important tool in clinical practice. Liver volume is assessed primarily via organ segmentation of computed tomography (CT) and magnetic resonance imaging (MRI) images. The goal of this paper is to provide an accessible overview of liver segmentation targeted at radiologists and other healthcare professionals.

**Methods:**

Using images from CT and MRI, this paper reviews the indications for liver segmentation, technical approaches used in segmentation software and the developing roles of liver segmentation in clinical practice.

**Results:**

Liver segmentation for volumetric assessment is indicated prior to major hepatectomy, portal vein embolisation, associating liver partition and portal vein ligation for staged hepatectomy (ALPPS) and transplant. Segmentation software can be categorised according to amount of user input involved: manual, semi-automated and fully automated. Manual segmentation is considered the “gold standard” in clinical practice and research, but is tedious and time-consuming. Increasingly automated segmentation approaches are more robust, but may suffer from certain segmentation pitfalls. Emerging applications of segmentation include surgical planning and integration with MRI-based biomarkers.

**Conclusions:**

Liver segmentation has multiple clinical applications and is expanding in scope. Clinicians can employ semi-automated or fully automated segmentation options to more efficiently integrate volumetry into clinical practice.

**Teaching points:**

• *Liver volume is assessed via organ segmentation on CT and MRI examinations.*

• *Liver segmentation is used for volume assessment prior to major hepatic procedures.*

• *Segmentation approaches may be categorised according to the amount of user input involved.*

• *Emerging applications include surgical planning and integration with MRI-based biomarkers.*

**Electronic supplementary material:**

The online version of this article (doi:10.1007/s13244-017-0558-1) contains supplementary material, which is available to authorised users.

## Introduction

Segmentation refers to the process of delineating an organ of interest—typically on multiplanar computed tomography (CT) or magnetic resonance imaging (MRI)—for volumetric or morphological analysis. The liver is one of the most difficult organs to segment due to its highly variable shape and close proximity to other organs. In addition, the liver is subject to diverse pathologies that may modify its density, signal intensity or distort its architecture. Examples include liver fat, iron deposits, fibrosis and tumours.

**Fig. 1:**
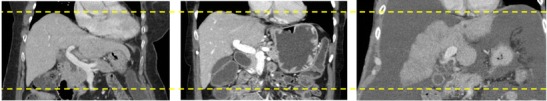
Variability of liver shape and size. Livers of different shape and volume may have similar cranial-caudal length, as demonstrated with these examples of three different patients. This observation highlights the limitation of reporting a one-dimensional measure of length, a well-entrenched practice, as a surrogate measure of liver volume

Although physical examination has long been used to detect liver size [1], the variability of liver sizes among patients limits its ability to detect pathology. As seen in Fig. 1, livers of similar cranial-caudal length may have markedly different volumes. Standard liver volumes can be calculated from the patient’s body surface area or mass using the formulas proposed by Vauthey et al. [2] in 2000. However, these formulas are limited by subject demographics (healthy individuals) and by their modest correlation to liver sizes calculated by more advanced forms of volumetry [3].

The era of modern imaging technology has offered new and more accurate tools to estimate liver volume. CT volumetry of the liver was first performed on cadavers by Heymsfield et al. [4] in 1979 and was shown to be accurate within 5% of water displacement volumetry. Today, liver volume can be calculated from both CT and MRI examinations. CT is more commonly used due to its greater accessibility, higher spatial resolution, robustness and short acquisition time [5–7]. MRI, conversely, offers multiple contrast mechanisms and the ability to assess vascular and biliary anatomy in addition to parenchymal pathology [8]. MRI also minimises the risk of nephrotoxicity and eliminates concerns of radiation exposure [8].

Liver segmentation has emerged as the preferred technique of liver volumetry. Liver segmentation involves identifying the voxels belonging to liver parenchyma on CT or MRI images via the generation of multiple segments. There are many approaches to segmentation that involve varying amounts of operator input, each with their own advantages and disadvantages.

The goal of this paper is to provide clinicians with an accessible primer for liver segmentation. We will first discuss the major indications for performing liver segmentation for volumetry. Next, we will provide an overview of the various segmentation techniques available. Finally, we will discuss the emerging clinical applications of liver segmentation.

## Indications for liver volumetry

### Future liver volume prior to major hepatectomy

**Fig. 2:**
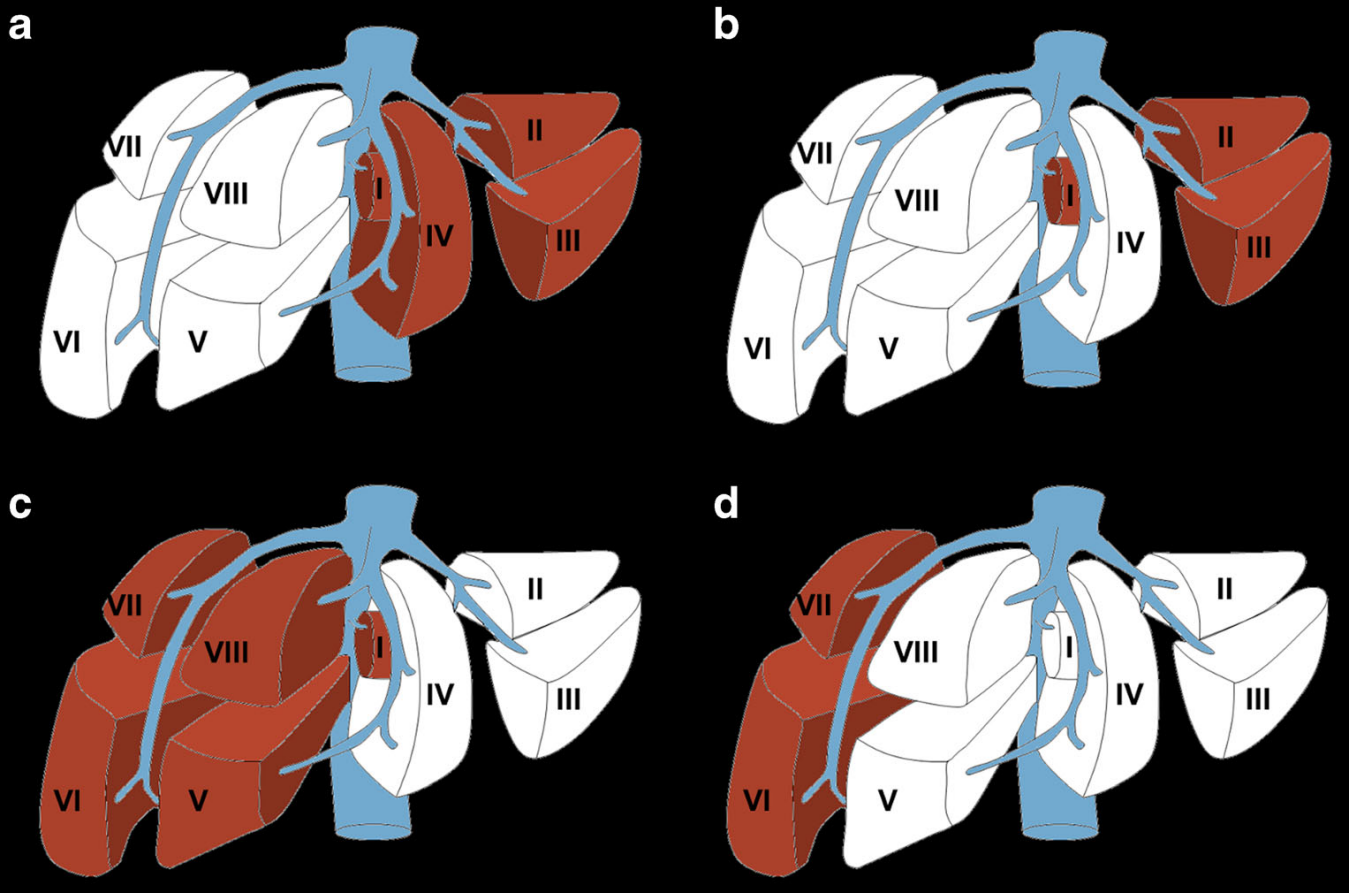
Types of major hepatectomy. White segments are planned for surgical resection. **a** Complete right hepatectomy. **b** Extended right hepatectomy. **c** Complete left hepatectomy. **d** Extended left hepatectomy. Figure adapted from the Brisbane 2000 Terminology of Liver Anatomy and Resections [12, 13]

Liver volumetry is indicated in patients undergoing major hepatic resection [9], defined as resection of four or more segments according to the Couinaud classification [10]. Most hepatectomies are performed for the treatment of neoplasms, including primary liver cancer (e.g. hepatocellular carcinoma and cholangiocarcinoma), liver metastases (e.g. from colorectal cancer) or certain benign tumours (e.g. giant haemangioma, adenoma, cystadenoma) [11]. Hepatectomies may also be performed for the management of localised abscesses (pyogenic, amoebic) or after trauma to the liver or biliary system. Different types of major hepatectomies—including left and right complete and extended hepatectomies—are outlined in Fig. 2 [12, 13]. There has been a recent rise in the number of extended hepatectomies being performed as definitions of resectability have expanded; extended right hepatectomies are currently the most common form of hepatectomy, representing 70% of all hepatectomies [14, 15].

Liver volumetry is a useful clinical tool for patients—both with and without underlying liver disease—undergoing major hepatic resection [14, 16]. The total liver volume (TLV) and the future liver remnant (FLR)—the amount of liver that would be left post-resection—are measured. FLR volume has been shown to be an indicator of both post-operative liver function and clinical outcome; it is also one of the only independent predictors of post-operative liver dysfunction [14].

**Fig. 3:**
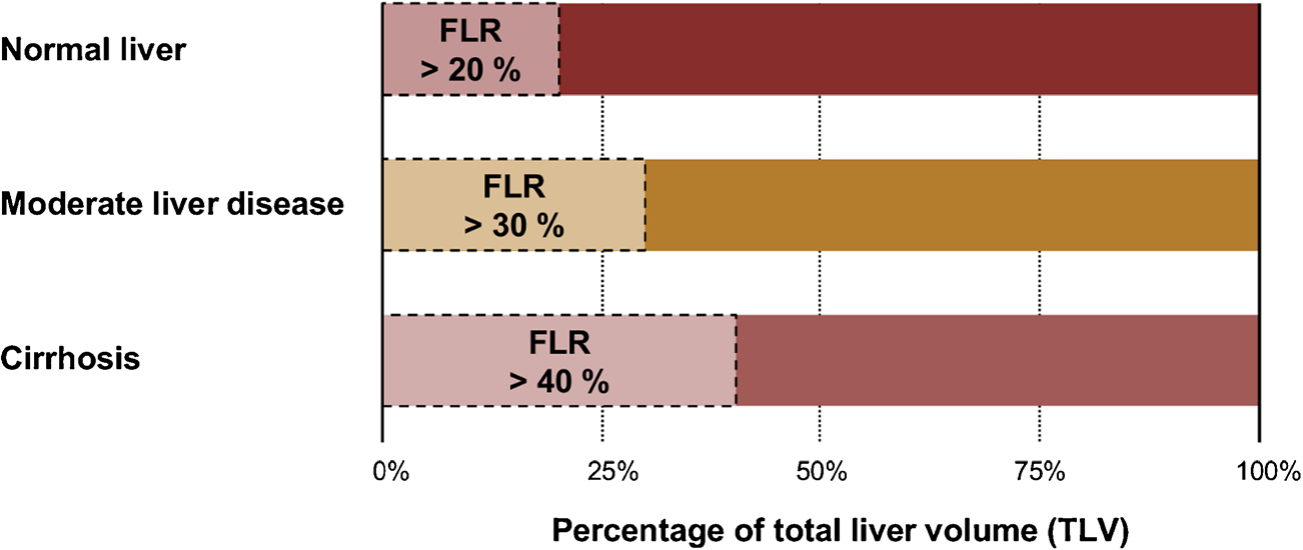
Schematic of functional liver remnant (FLR) over total liver volume (TLV) ratio prior to hepatectomy. To be considered safely resectable prior to hepatectomy, the FLR/TLV ratio must be >20% in underlying normal livers, >30% in moderately diseased livers and >40% in cirrhotic livers

**Fig. 4:**
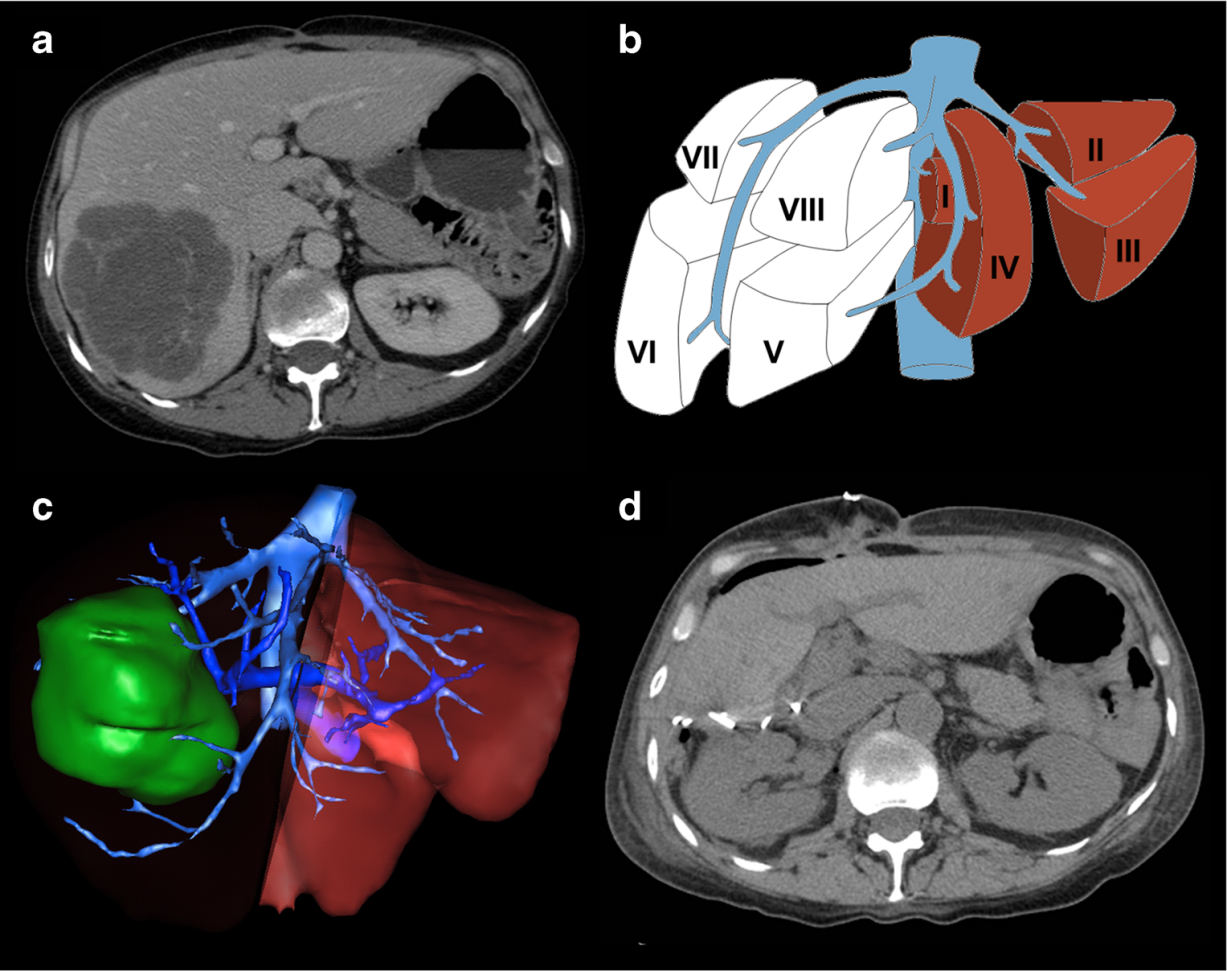
Future liver remnant volume calculation in normal liver prior to right hepatectomy. **a** Axial enhanced CT image showing colorectal liver metastasis involving right posterior segments (VI and VII). **b** Resection diagram shows the intended complete right hepatectomy surgery planned. **c** Three-dimensional rendered image showing surgical planning for complete right hepatectomy. FLR/TLV ratio was estimated to be 33%. **d** Axial unenhanced CT image of the same patient shortly after complete right hepatectomy. Actual FLR/TLV ratio was calculated to be 36%. Figure courtesy of Dr. Vandenbroucke-Menu; created with 3DVSP (IRCAD, Strasbourg, France)

In patients with otherwise normal livers, the FLR/TLV ratio should be >20%; in patients with moderately diseased liver, the ratio should be >30%; finally, in patients with cirrhosis or fibrosis, the ratio should >40% (Fig. 3) [17]. Moderate liver disease has been defined as liver steatosis secondary to extensive chemotherapy. Certain systemic chemotherapies directly induce hepatic steatosis and sinusoidal obstruction syndromes resulting in fatty liver parenchyma [1]. The extent of the liver pathology may be related to other patient specific factors such as obesity, diabetes and presence of metabolic syndrome [2]. These factors may also impact a given patient’s tolerance of surgery. Therefore, the aforementioned limits should be viewed as somewhat flexible. An example of FLR/TLV ratio calculation prior to hepatectomy and the post-operative result is demonstrated in Fig. 4 for a normal liver. Patients who are not candidates for hepatectomy based upon the aforementioned criteria are at increased risk for post-operative liver dysfunction and may undergo pre-operative portal vein embolisation (PVE).

### Portal vein embolisation (PVE)

Portal vein embolisation (PVE) is performed by an interventional radiologist prior to major hepatectomy to maximise viable FLR. In PVE, portal vein branches that supply liver segments to be removed during hepatectomy are embolised. This results in the redistribution of blood flow towards the non-embolised segments, promoting liver hypertrophy and increasing anticipated FLR. Patients who have undergone PVE demonstrate improved liver function post-extended hepatectomy compared to patients who have not [2, 16].

**Fig. 5:**
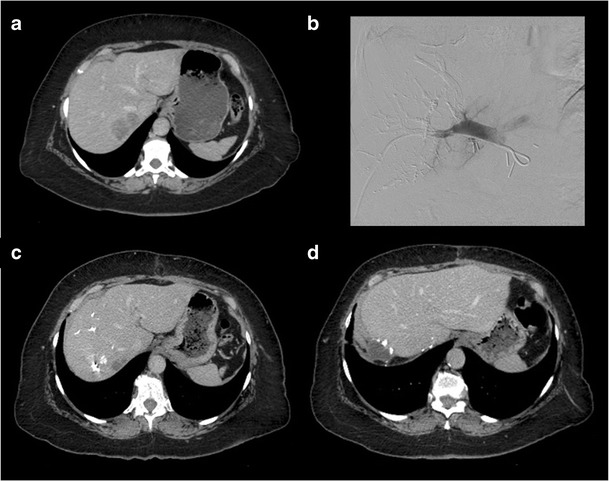
Portal vein embolisation prior to right hepatectomy. **a** Axial enhanced CT image shows colorectal liver metastasis involving segments V, VI, VII (only VII shown). **b** Final portogram of embolised portal vein branches in segments V through VIII using a Lipiodol-glue mixture. **c** Axial enhanced CT image obtained 1 month after right PVE showing hypertrophy of future liver remnant. **d** Axial enhanced CT image of the same patient after right hepatectomy

PVE is indicated when an increased post-hepatectomy FLR volume is required for adequate post-operative hepatic function. This may be due to underlying liver disease or the extent of the resection planned. Individuals who are expected to have suboptimal FLR/TLV ratios (as per Fig. 3) are candidates for PVE [17]. Liver volumetry is generally re-performed 3–4 weeks post-PVE to assess the extent of hypertrophy of the FLR prior to surgery [16]. Patients who have reached a standardised FLR (FLR divided by total liver volume as calculated using the Vauthey equations) of 20% or a degree of hypertrophy of 5% by this time have more favourable post-hepatectomy outcomes [18]. However, evidence has emerged that an increase in liver function—as measured by ^99m^Tc-labelled mebrofenin HBS with single photon emission tomography—may precede the increase in FLR [19]. Furthermore, the rate of hypertrophy (not just the degree) may also be an important indicator of pre-operative readiness [20]. This may explain why certain patients may tolerate hepatectomy earlier than 3 weeks post-PVE. On the other hand, other practitioners recommend waiting up to 6 weeks [21]. An example of a patient who underwent PVE prior to right hepatectomy is shown in Fig. 5.

### Associating liver partition and portal vein ligation for staged hepatectomy (ALPPS)

In patients with more extensive, rapidly expanding, or bilobar disease, associating liver partition and portal vein ligation for staged hepatectomy (ALPPS) has gained popularity. ALPPS is as two-staged surgical procedure that may offer rapid and major FLR hypertrophy and a decreased risk of post-operative liver failure compared to PVE followed by hepatectomy [22]; however, it is also associated with greater peri-operative morbidity and mortality [23].

In the first stage of the procedure, a total resection of tumours from the future FLR is performed (if applicable), followed by ligation of the portal vein that supplies the liver to be removed and transection of the liver parenchyma. Both the FLR and remaining segments are left in situ; the arterial and biliary systems belonging to the portion of the liver to be removed are preserved for synthetic function and to avoid liver necrosis before the second stage. After sufficient hypertrophy of the FLR is achieved (generally within 1 week), the abdomen is reopened for the second stage and the deportalised liver is removed [24].

Liver volumetry is performed prior to each stage of the procedure. ALPPS is indicated based on anticipated FLR volume: in general, the procedure may be offered when pre-operative volumetry predicts an insufficient FLR associated with major liver tumour and/or additional liver pathology (including chemotherapy-induced damage, fibrosis, cholestasis or macrosteatosis) [25]. Volumetry is repeated prior to the second stage to ensure sufficient FLR hypertrophy; a 30% standardised FLR based on the Vauthey equations is generally expected before proceeding to the second stage (although greater hypertrophy has been noted in the literature) [22, 25].

### Pre-transplant volumetry

Living-donor liver transplants are increasingly being performed given the rising demand for transplant and the diminishing availability of cadaveric livers [26]. As such, opportunities are being sought to improve donor and recipient outcomes. In the paediatric recipient population, for example, it has been shown that transplant of the just the left lateral segment from a living adult donor is sufficient for adequate recipient liver function; however, this is not the case in the adult recipient population [27].

**Fig. 6:**
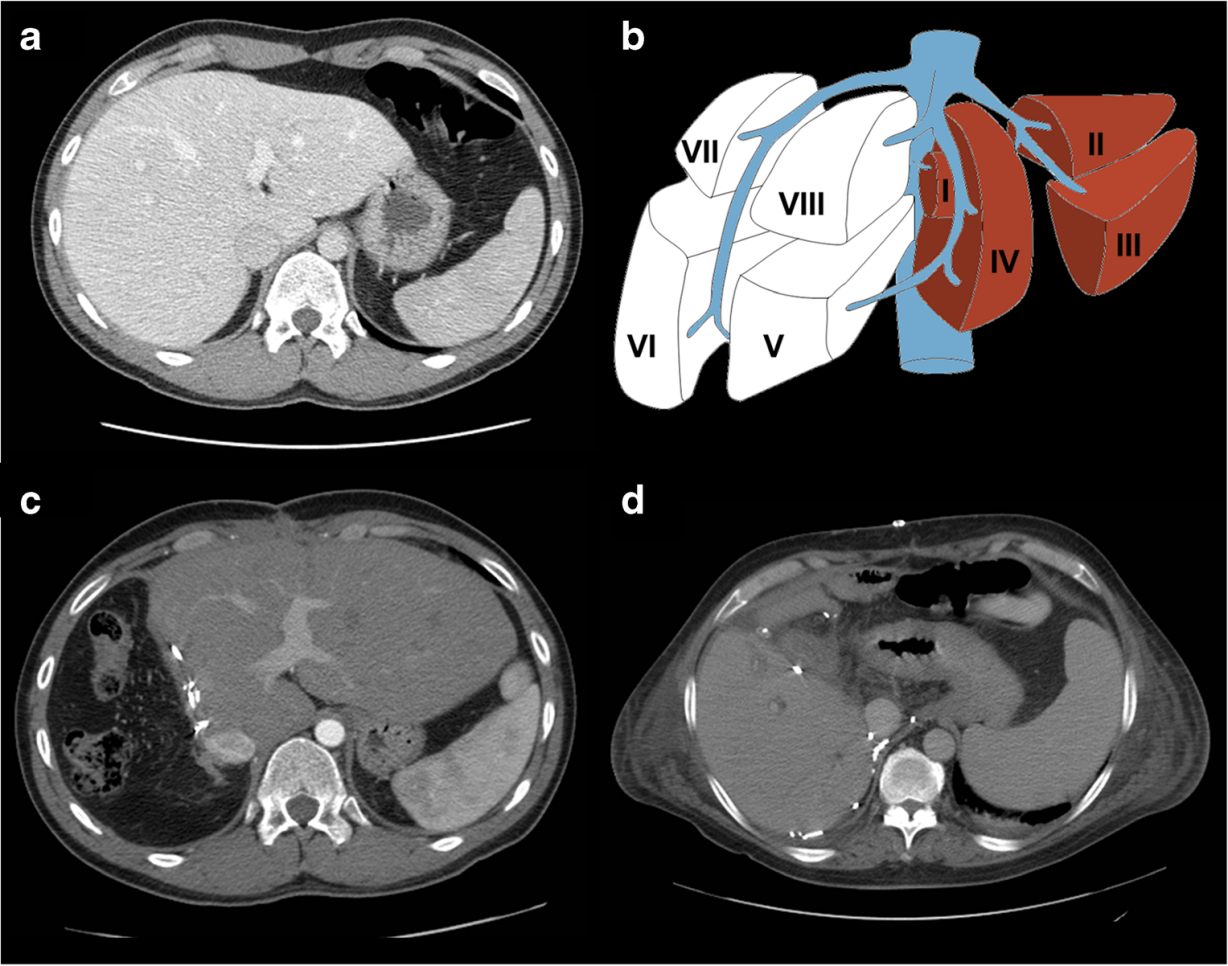
Size incompatibility after living donor liver transplantation: both the donor and the recipient suffered transient hepatic insufficiency. **a** Axial enhanced CT image of a 26-year-old living liver donor. The total liver volume (TLV) was 1,754 mL. The donated liver volume was 980 mL and the residual liver volume was 774 mL (44.2% of the TLV). **b** Diagram showing the intended right split liver surgery planned for living donor liver transplantation. **c** Post-liver transplantation axial enhanced-CT image showing hypertrophied left liver of the donor. **d** Post-liver transplantation axial enhanced-CT image of a 53-year-old man who was the recipient of the right liver transplant. Although pre-transplant volumetry calculations seemed to indicate that the liver size was appropriate, the patient still developed small-for-size syndrome requiring ligature of the splenic artery. The cause was likely multifactorial. The donor developed transient biological hepatic insufficiency that resolved with supportive management

Pre-transplant volumetry is indicated to ensure appropriate graft size for successful donor and recipient outcomes. To optimise donor graft survival, an FLR/TLV ratio of 30–40% is recommended [28, 29]. The ratio of graft size to standard liver volume (from body surface area) of the recipient should be over 50% [30]; alternatively the ratio of graft size to body weight of the recipient should be over 0.8–1.0%. Inadequately sized grafts may be functionally insufficient and may result in small-for-size syndrome, a potentially fatal condition of hepatic insufficiency that may require re-transplant [31]. Small-for-size syndrome is multifactorial, however, and may develop even when appropriate pre-operative volumetry is performed (*see* Fig. 6).

In the case of right-lobe liver donor transplantation, there is a dilemma surrounding the inclusion of the middle hepatic vein (MHV) in the donated graft. The inclusion of the MHV in the graft is required for adequate venous drainage in the recipient, but may lead to congestion of segment IV in the donor liver [32]. To strike a balance, the MHV is therefore usually transected proximal to a major segment IVb hepatic vein. This makes pre-operative volumetry along with vascular assessment advantageous for positive patient outcomes. Please see the “Vascular subsegmentation” section for further information.

## Segmentation techniques

**Fig. 7:**
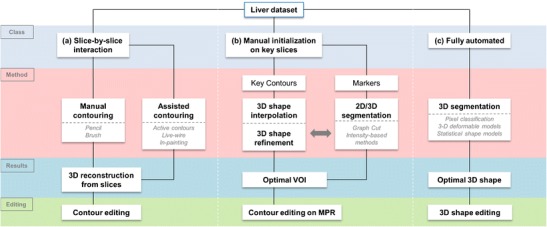
Workflows of various segmentation strategies. The schematic breaks down liver segmentation methods into truly manual, contour optimisation, semi-automated, and fully automated workflows. Most workflows require a combination of two-dimensional or three-dimensional initialisation, refinement and editing techniques. *VOI* volume of interest, *MPR* multi-planar reconstruction

In this section, the workflow of manual, semi-automated, and fully automated segmentation strategies will be described (Fig. 7), with a summary of the strengths and limitations of each technique.

## Manual segmentation

Manual liver segmentation relies heavily on user-interaction to perform segmentation. Manual segmentation is performed via the contouring of pixels along the boundary of the liver or the in-painting of the liver parenchyma on sequential CT or MR slices. Once the liver has been identified on each slice, post-processing software is used to generate liver volume. Early manual segmentation approaches used very basic tools such as a pencil, spline widget or paintbrush. Newer manual techniques use algorithms to optimise contouring or in-painting; despite this rudimentary level of automation, these techniques may still be considered manual.

**Fig. 8:**
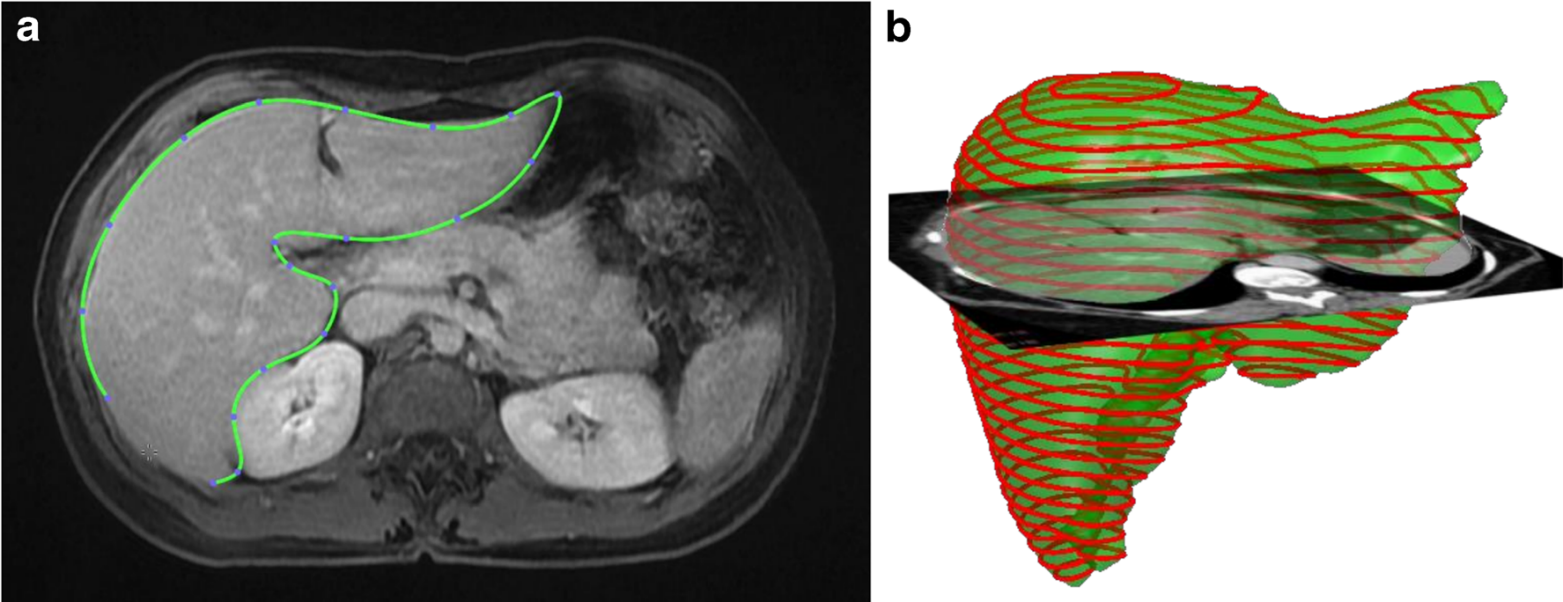
Manual segmentation of the liver. **a** Manual segmentation of the liver performed by contouring of pixels of the liver boundary on CT image. Image obtained using Osirix image post-processing software (Osirix Foundation, Geneva, Switzerland). **b** Volume of the liver is obtained based on pixel size and slice spacing [13]

To perform manual segmentation via contouring, axial CT or MR images are saved as Digital Imaging and Communications in Medicine (DICOM) files and loaded to post-processing software. The image analyst then uses a cursor to position nodes along the liver boundary (*see* Fig. 8a or the supplementary material for an animation). Vessels enclosed by the parenchyma are included, while those that are adjacent to the liver—such as the portal vein and the inferior vena cava—are excluded. The total number of pixels within the bounded area provides the cross-sectional area for a given slice (Fig. 8b). Each slice area is then multiplied by the slice thickness and the resulting volumes are summed to provide the total liver volume. Similar inclusion and exclusion parameters are used for in-painting, although in this case the parenchyma is swept-over by the user to select areas of interest within the liver [33].

There are many drawbacks of manual segmentation. There is inherent intra-observer and inter-observer variability given its subjectivity. Variability is also be introduced by the sharpness of liver boundaries, window level settings, and computer monitor settings [34]. Manual segmentation is also time-consuming and may take up to 90 minutes for one patient [35]. As a result, manual segmentation is not suited for a busy clinical practice in high volume settings. Examples of assisted contouring and in-painting techniques used during manual segmentation are addressed below.

### Assisted contouring

#### Active contours

**Fig. 9:**
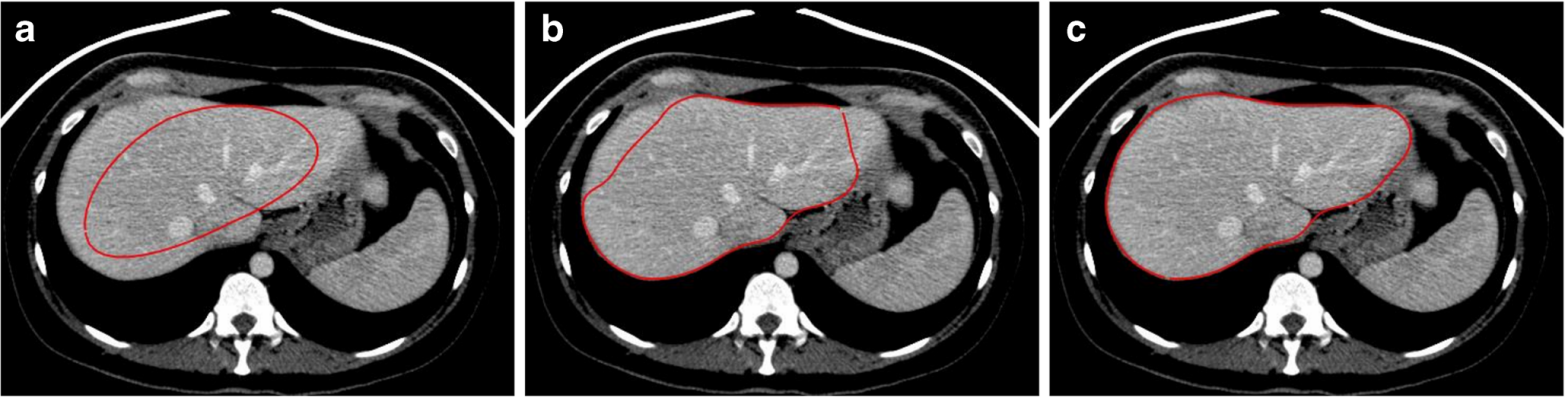
Active contours technique. **a** Image analyst roughly contours the liver using a cursor. **b** Contour evolves based on salient image features. **c** The contour snaps to the true liver contour [13]

In the active contour approach, the image analyst draws a rough contour of the liver with the cursor. These contours, also called snakes, are then tested with an interactive algorithm that either forces the snake to collapse or expand based on the data-set provided by the image. Ultimately, the contour should snap to the outer borders of the liver (see Fig. 9 or the supplementary material for an animation) For optimal segmentation, the image must be adequately preprocessed and the original contour must be close to the liver boundary. This will prevent the algorithm from leaking into nearby organs or falling into a local minimum. Of note, the active contours technique is the basis for software SliceOmatic® created by Tomovision [36].

#### Livewire

**Fig. 10:**
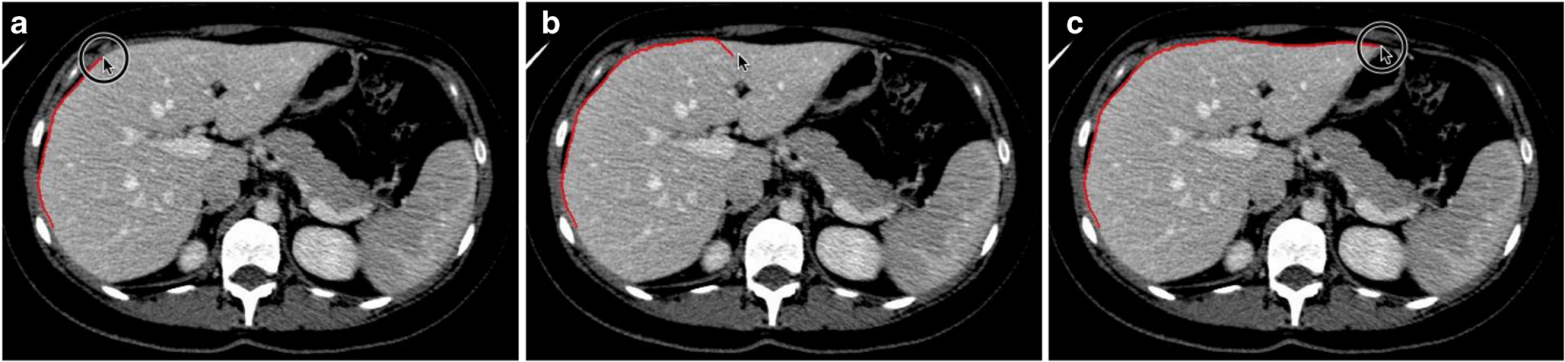
Livewire technique. **a** User sets the “seed point” by clicking on the liver boundary. **b** As the cursor is moved, the boundary behaves like a livewire, connecting the seed point to the cursor. **c** The free point is placed along the liver boundary, and a minimal cost path is generated [13]

In the livewire approach the image is interpreted as a weighted graph [37]. The pixels are represented by graph vertices. Adjacent pixels are connected by graph edges; the cost of connection between these vertices is represented by the weight of these edges. The user clicks on the boundary to create a “seed point” and the possible minimal cost pathways to all other points on the image are calculated. Then, the user chooses another boundary point, which is called the “free point”. The boundary of the liver then behaves like a livewire, connecting the seed point with the free point via a minimal cost path along the liver edge (see Fig. 10 or the supplementary material for an animation). The two-dimensional (2-D) livewire technique serves as the basis for the software HepaVision® created by MeVisLab [7, 13].

#### Shape interpolation

Shape interpolation allows the user to interpolate a complete three-dimensional (3-D) shape by tracing a limited number of contours. This reduces the number of images that must be contoured to a few key slices. This technique can be combined with the livewire approach to further optimise each of the interpolated contours. This assisted contouring technique serves as the basis for the 3-D deformable models technique, which will be discussed in the section on fully automated segmentation.

### Assisted in-painting

#### SmartPaint

SmartPaint employs a paintbrush paradigm. The user to sweeps over the liver parenchyma and the algorithm selectively sticks to certain regions, while avoiding others. This identifies the underlying voxels as belonging to either the object (i.e. liver) or its background. The segmentation is updated in real-time to provide immediate user feedback updating the segmentation. Like with assisted contour techniques, once all of the voxels belonging to the liver are accounted for, the volume of the organ may be calculated [33].

## Semi-automated segmentation

Semi-automated segmentation techniques require coarse initialisation from the user; the algorithm then provides the majority of the optimisation. These techniques often rely on a combination of interactions. Examples include intensity-based techniques and graph cut.

### Intensity-based techniques

**Fig. 11:**
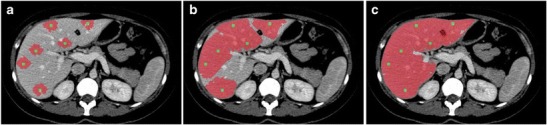
Seeded region-growing technique. **a** Seeds are positioned inside the regions of interest by user. **b** Pixels are iteratively aggregated if their intensity is similar to those already tagged. **c** The liver parenchyma is segmented

Intensity-based techniques classify pixels and their neighbours according to intensity or texture. For example, seeded region growing is an intensity-based technique where “seeds” are positioned by the user in the liver parenchyma (see Fig. 11 or the supplementary material for an animation) [38]. The pixels then iteratively aggregate if their intensity matches that of those already tagged. As a result, intensity-based techniques perform very well on homogenous livers. However, since there is no shape control, intensity-based techniques may result in leakage of the seeded area or rough edges. This is particularly problematic in diseased livers where substantial user interaction may be required to achieve desired results. Furthermore, intensity-based techniques are ill-suited MR segmentation of the liver due to the greater heterogeneity of the parenchyma on this modality [39].

### Graph-cut

In the graph-cut technique, the user roughly paints some foreground (i.e. the liver) and background pixels (i.e. peri-hepatic structures). Based on graph analysis and optimisation, a cut is then performed to separate the foreground and background areas in the most homogeneous regions. This isolates the liver on the given image slice for volumetry [40] (see supplementary material for an animation of this technique).

## Fully automated segmentation

Fully automated segmentation techniques require no, or negligible, user input for typical datasets. However, they may require manual adjustment for pathological or unusual cases.

### Statistical shape models (SSMs)

**Fig. 12:**
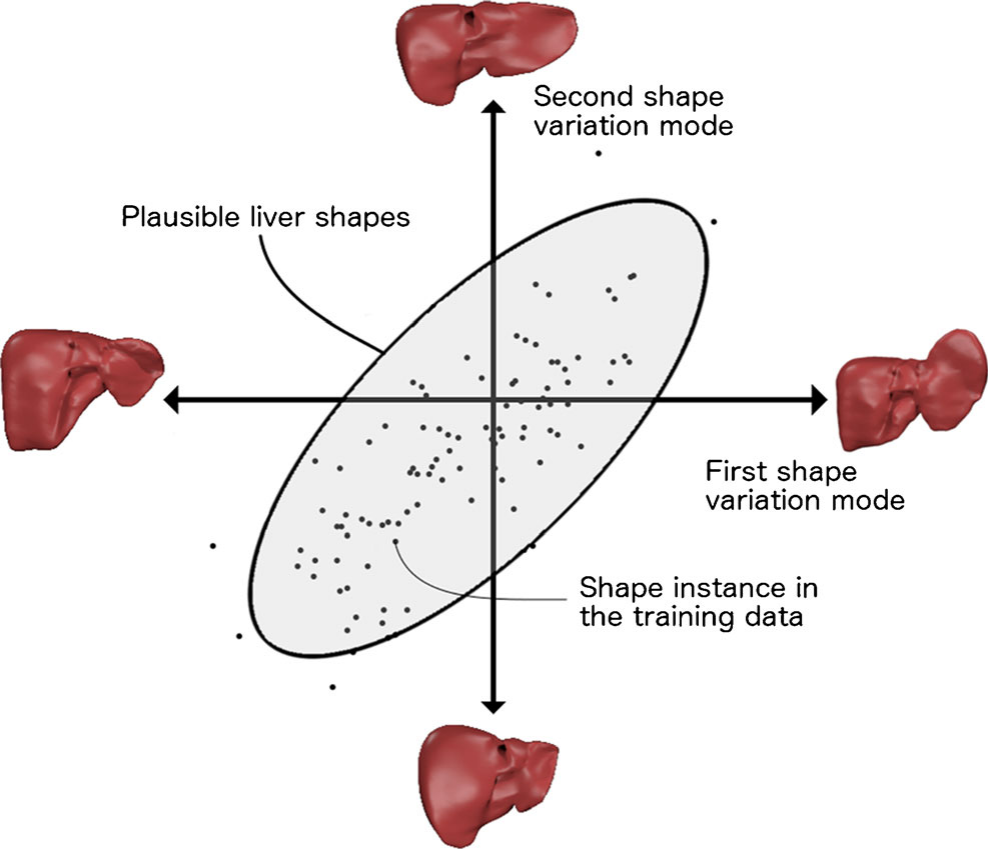
Statistical shape models. To restrict the segmentation to a set of admissible liver shapes, a shape database is compiled, from which any new liver shape is expressed by a set of parameters called modes of variation. The various modes of variation (roughly 30 modes) are adjusted to fit the liver shape on image features. Statistical shape models impose hard restriction on the segmentation outcome by integrating prior shape. However, training data cannot capture all variations and therefore are sometimes too limiting to accurately model specific livers [13]

Statistical shape models (SSMs) use global shape priors (i.e. multiple geometric representations of the liver) to generate segmentations that do not deviate from a reasonable liver shape. This creates a hard constraint on liver morphology from which the segmented liver is not permitted to deviate, preventing segmentation leakage. Early studies used a single global shape prior [41]; however, this had limited versatility, given the variability of liver shape among patients. SSMs expand the range of admissible liver shapes [42]. In this approach, the image data is used to deform a surface mesh parameterised by certain admissible shapes (Fig. 12). Typically, about 30 shapes are used to establish the dataset.

SSMs offer an advantage over the previously discussed segmentation techniques because of the precise modelling of shape variations, which can be used to regularise the liver’s appearance. This technique can produce accurate segmentation despite signal noise and retain robustness despite proximity of the liver to similarly appearing organs [43]. However, SSM requires 30–75 segmented liver shapes to generate a training dataset from which the main shape variations are derived. Furthermore, the resulting model may be too constraining if a patient’s liver shape is not adequately represented in the training data. This may result in limited application to pathological or post-operative livers.

### 3-D deformable models

A 3-D surface mesh of the liver may be iteratively deformed to fit the liver boundaries by interpolating a surface between a few sparse contours. This technique is based upon shape interpolation, which was discussed in manual segmentation. For each vertex of the generated mesh, matched features corresponding to the liver boundary are identified in the patient dataset. This mesh may then be subject to a non-rigid registration scheme [44], which deforms the shape towards the liver boundary while preserving surface smoothness [35, 45]. If carefully tuned, a simple sphere can initiate the process. Deformable models may also be derived completely independently of user input (*see* “Advanced segmentation” below).

### Pixel classification approaches

A wide range of segmentation techniques use quantitative image texture features to train classification algorithms. Examples include support vector machines (SVMs) [46] and random forests [47]. Even though such features have a higher discriminative power than the intensity based techniques, they can lead to coarse segmentation and leakage. Recently, convolutional neural networks were proposed in the setting of liver segmentation, where quantitative features were learned rather than being handcrafted. This technique allowed for the segmentation of heterogeneous livers obtained using different scanners and protocols in under 100 seconds [48].

## Advanced segmentation techniques

The previously presented segmentation techniques are seldom used on their own. Advanced segmentation strategies often combine various segmentation techniques. For example, pixel classification can be combined with graph-cut optimisation, SSM [47] or probabilistic models [49, 50] for robust automated segmentation. SSM may also be used as a robust initialisation phase in combination with a 3-D deformable model to better account for the natural heterogeneity between and within livers [51].

## Summary

A summary of the advantages and disadvantages of various segmentation techniques illustrated in the previous sections is provided in Table 1.

**Table 1:**
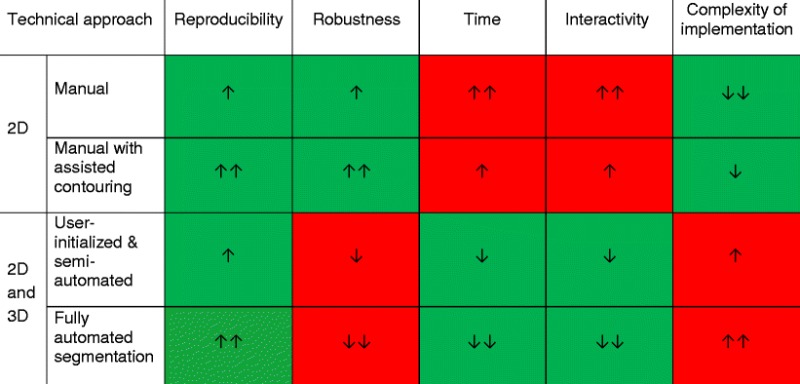
Summary of advantages and limitations of various segmentation methods [13]

Note: Green cells indicate desirable features, whereas red cells indicate limitations. One arrow indicates a minor feature, whereas two arrows indicate a major feature. The direction of the arrow refers to increase or decrease in the specific parameter.

## Alternatives to liver segmentation for volumetry

Alternative methods of volumetric analysis, such as stereology, merit mention. Stereology is a volumetric technique that employs statistical methods to calculate liver volume, whereby a sample of pixels is selected on individual liver slices to calculate the entire liver volume. This sample can be taken using a variety of methods, such as grid-based sampling method (the Cavalieri method). In this method, a regular grid is placed over cross-sectional images; each time the liver touches a pre-selected structure (e.g. right lower corner of a square), it is counted in the volumetric calculation [52]. The calculated cross-sectional area is then summed across slices to calculate the volume [53]. Advantages of such a method include speed, cost-effectiveness, multimodal applicability (CT, MRI, ultrasound) and the ability to perform subsegmentation (lesions, vascular structures, specific liver segments) [52]; disadvantages may include variation based on slice thickness and slightly decreased accuracy [53].

## Imaging requirements

Liver segmentation methods can generally be applied to any modality, sequence or vascular phase. The success of a given technique, however, depends upon the quality of implementation, which is the extent to which the technique has been trained to initiate and regularise segmentation for a given image type. This depends not only on the technique itself but also on the appearance model used in the study. The appearance model (i.e. what the technique classifies as liver) must be well adapted for the modality, sequence, and vascular phase to discern liver tissue from adjacent structures for optimal segmentation.

CT is generally preferred over MRI for segmentation because of higher spatial resolution, isotropic voxels, robustness (does not require long breath-holds) and calibrated Hounsfield units (whereas MRI provides arbitrary signal intensity). The portal venous phase also tends to be popular as it enhances the liver parenchyma.

For MRI, the contrast agents gadoxetate disodium (Primovist, Eovist; Bayer Healthcare, Leverkusen, Germany) and gadobenate dimeglumine (MultiHance; Bracco Diagnostic, Milan, Italy) can help improve liver segmentation. Both are gadolinium-based contrast agents that provide hepatobiliary phases during MRI occurring 20 minutes and 2 hours after contrast injection [54]. Imaging in the hepatobiliary phase reveals strong uptake in the normal liver, which may accentuate the contrast with non-hepatic tissue (e.g. vessels or focal liver lesions) and adjacent organs. The hepatobiliary uptake is roughly 50% of the injected dose of gadoxetate disodium and 5% of the injected dose﻿ of gadobenate dimeglumine [55]. The hepatospecificity of gadoxetate disodium increases the contrast between liver parenchyma and liver lesions or vascular structures compared to conventional contrast agents and other imaging modalities [54, 56, 57].

The increased contrast with gadoxetate disodium-enhanced MRI has been exploited for liver segmentation purposes. For example, Grieser et al. [58] showed that liver segmentation of Gd-EOB-enhanced T1-weighted 3-D gradient-recalled-echo images using threshold-based techniques are both accurate and time-saving. The authors showed that the performance of this approach can be further improved by increasing flip angle. Fernandez-de-Manuel et al. [59] proposed a multimodal non-rigid registration framework combining gadoxetic acid-enhanced MRI and contrast-enhanced CT images to characterise liver lesions. Despite these benefits, these contrast agents will not likely take the place of the portal venous phase due to the latter’s specificity and utility for overall liver volumetry and vascular subsegmentation [59].

## Segmentation pitfalls

There are several sources of error that must be accounted for during the usage of automated segmentation techniques. The following section outlines sources of error associated with imaging modality and patient anatomy.

### Error linked to modality

The imaging modality used for segmentation has a direct impact on the quality of the features extracted from the images. With poorer feature extraction comes a higher risk of segmentation error and divergence. Specific acquisition parameters and imaging artefacts can affect segmentation results and represent potential sources of error.

Breath-holding can be used during both CT and MRI to limit the effects of respiratory motion on the quality of images. CT is less affected by motion due to rapid acquisition times. MRI requires longer acquisition times to achieve adequate spatial resolution and signal-to-noise ratio. Slice thickness can be increased to improve *z*-axis coverage in patients with limited breath-holding capacities. However, this results in partial volume effects when the voxels at the interface of two structures with varying signal characteristics must be averaged. In addition, the large spaces between voxels must also be interpolated, increasing volumetric error.

Slice thickness impacts liver volumetry results [30, 53, 60]. In general, thinner slices tend to be more accurate for estimation of liver volume using MRI [53]. Reiner et al. [60] suggested that 8-mm slices on MRI and 6-mm slices on CT provided the best compromise between volumetric precision and efficiency.

**Fig. 13:**
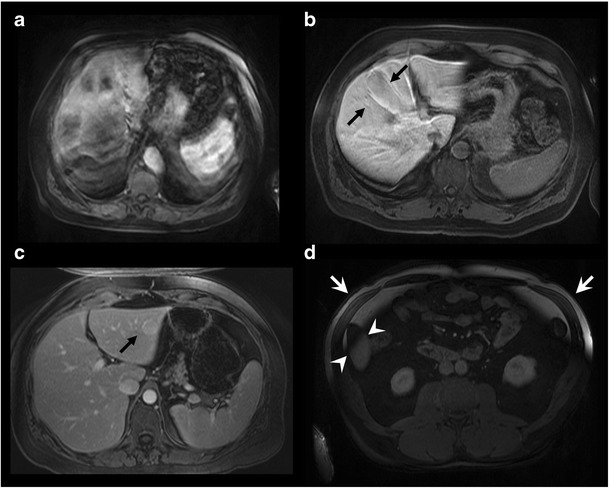
Imaging pitfalls which may degrade liver segmentation on MRI. Axial T1-weighted fat-saturated images with contrast injection depict the following artefacts: **a** Severe motion artefact. **b** Partial volume averaging of the liver parenchyma with the gallbladder (*arrows*). **c** Ghost artefact with the aorta (*arrow*). **d** Inhomogeneous fat saturation (*white arrows*) and fat-water swap in the liver (*arrowheads*) [13]

CT and MRI are both subject to artefacts. Metallic objects—such as surgical clips—cause streak artefacts on CT and susceptibility artefacts on MRI, which may both cause segmentation error. Other artefacts, such as motion, pulsation and partial ​volume averaging artefacts, may also interfere with segmentation accuracy [30, 61]. Figure 13 illustrates artefacts and imaging pitfalls commonly seen on MRI.

### Error linked to anatomy

**Fig. 14:**
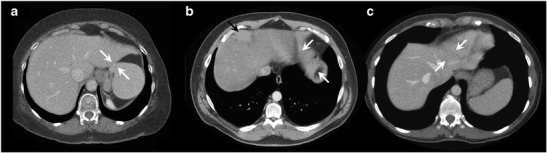
Imaging pitfalls which may limit liver segmentation on CT. **a** Axial enhanced CT image of a 62-year-old woman shows indistinct liver-spleen boundaries (*arrows*). **b** Axial enhanced CT image of a 47-year-old man depicts segmentation challenges caused by ill-defined and non-continuous borders found near the liver dome (*arrows*). **c** Axial enhanced CT image of a 73-year-old man shows partial volume averaging between the left liver and the heart (*arrows*) [13]

Normal and abnormal anatomical features may cause segmentation error. On CT, segmentation error often occurs at the interface of the liver parenchyma with the stomach, intercostal muscles, diaphragm, spleen and heart (Fig. 14). Error on CT and MRI may occur at low-contrast borders, adjacent to tumours, near vascular insertions and at the hepatic flexure [43]. Over-segmentation tends to occur at peri-hepatic organs, whereas under-segmentation tends to occur in areas of inhomogeneous density and at low-contrast liver boundaries [61]. Liver pathology—including steatosis, cirrhosis, malignancies, areas of ablation and polycystic disease—may distort the liver and cause an irregular or lobulated morphology which may also interfere with automated segmentation processes. These errors can be overcome with increased user interaction, either during the initialisation phase or with interactive correction tools.

## Emerging surgical needs and future directions

### Vascular subsegmentation

**Fig. 15:**
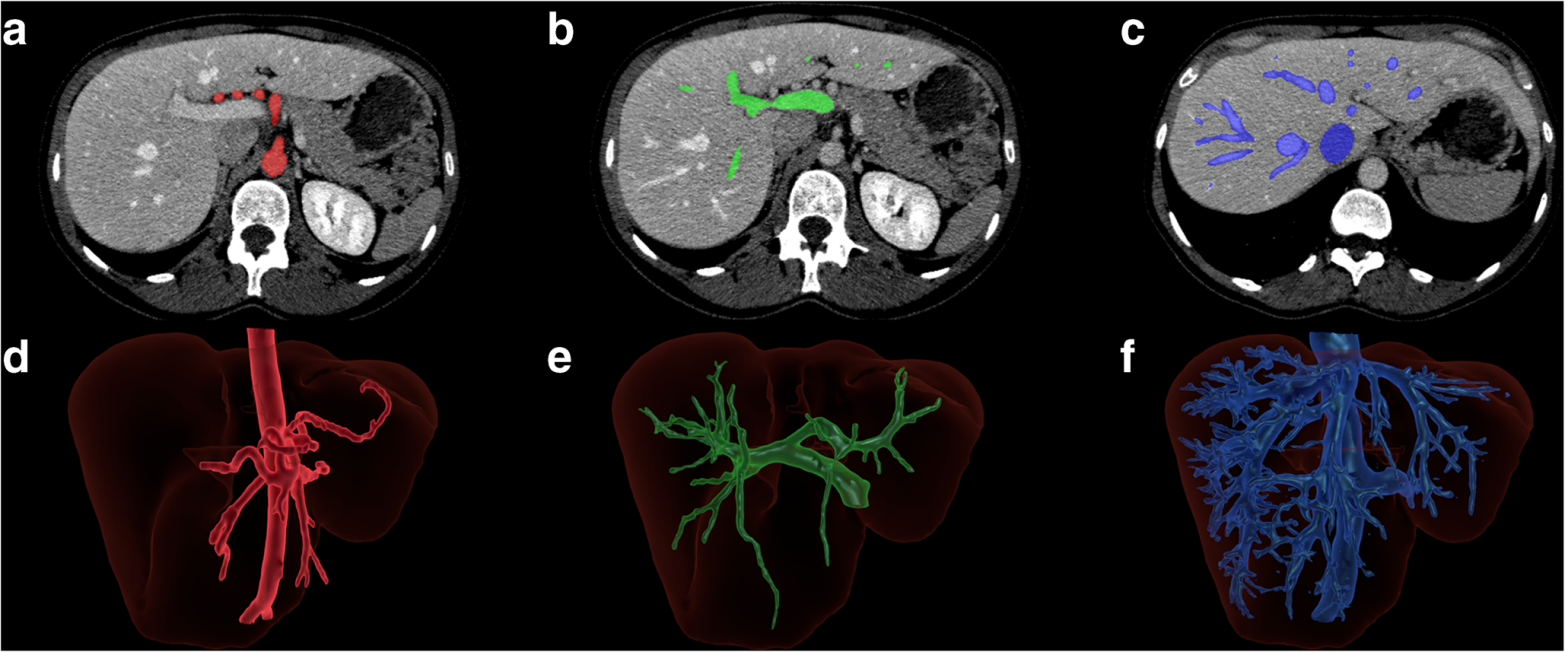
Liver subsegmentation according to vascular anatomy. Axial enhanced CT showing the segmented **a** hepatic arterial, **b** portal venous and **c** hepatic venous structures. Three-dimensional rendering of the same liver showing the corresponding segmented **d** arterial, **e** portal venous and **f** hepatic venous structures [13]

As previously mentioned, the standard Couinaud classification system does not take into account the numerous anatomical liver variants encountered in individual patients. In the near future, liver subsegmentation may be performed regularly according to vascular supply (i.e. portal veins and hepatic arteries) or drainage (i.e. hepatic veins) (Fig. 15). Such segmentation will provide surgeons crucial information regarding anatomical variants prior to hepatectomy or transplant.

### Surgical planning

**Fig. 16:**
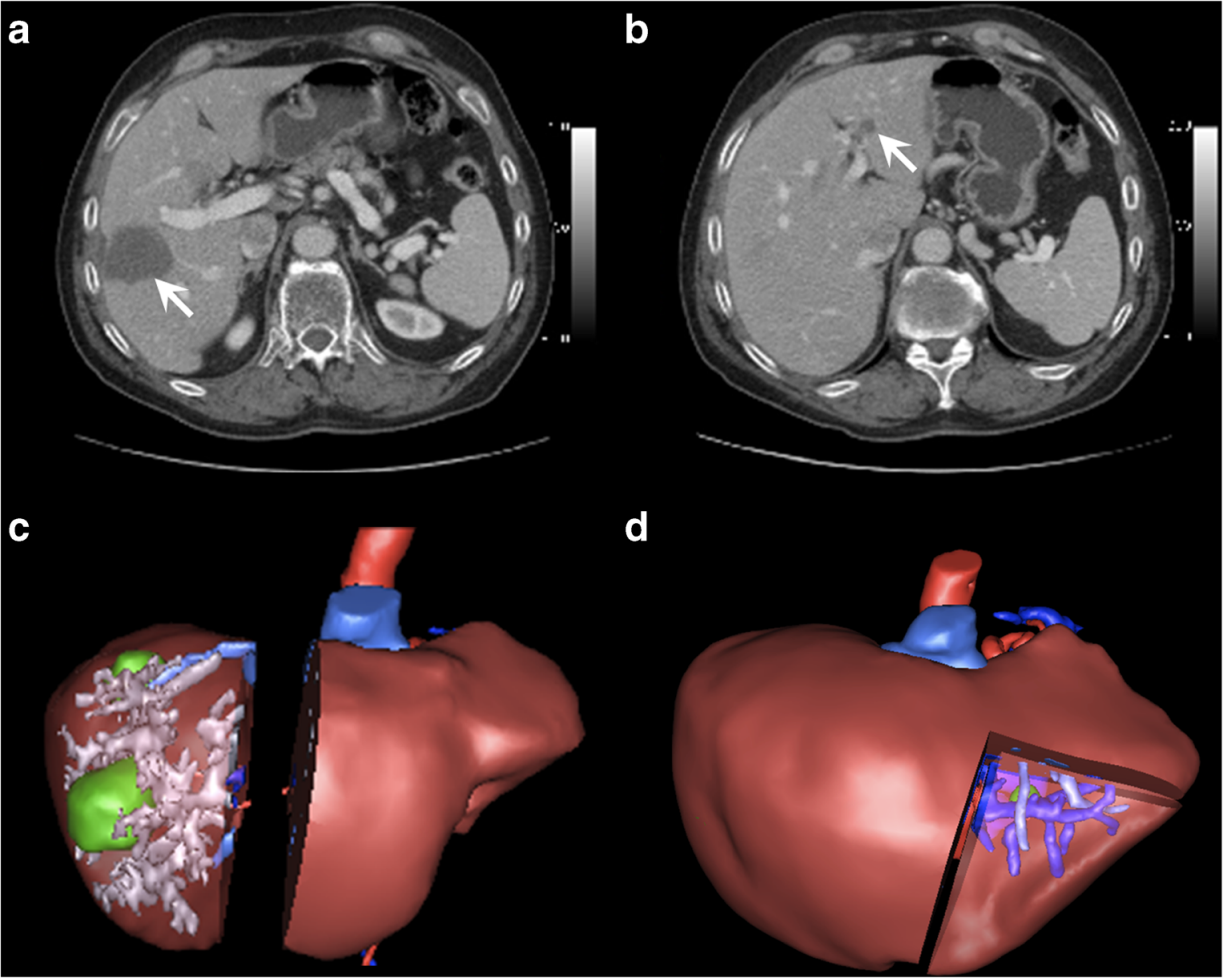
Virtual surgical planning. **a** Axial enhanced CT image shows a right liver metastasis centred in segment V (*arrow*). The patient also had a metastasis involving segment VII (not shown). **b** Axial enhanced CT image of a different patient shows a left liver metastasis in segment III (*arrow*). **c** Three-dimensional rendering image shows surgical planning for complete right hepatectomy including tumour and hepatic structures in patient from **a**. **d** Three-dimensional rendered image shows surgical planning for segmentectomy of segment III for patient in **b**. Residual hepatic liver volume after both procedures was estimated to be 27%. Right portal embolisation was thus performed before right hepatectomy. Figure courtesy of Dr. Vandenbroucke-Menu; created with 3DVSP (IRCAD, Strasbourg, France)

Commercially available segmentation solutions may provide virtual surgical simulation tools for hepatectomy or transplant. Using the segmentation techniques previously discussed, these solutions can integrate parenchymal and vascular segmentation tools for 3-D modelling (Fig. 16). These techniques can also be used to model lesions to exclude them from volumetric analysis. In general, semi-automated techniques are more successful in allowing the user to include or exclude the lesion based on the clinical context, as lesions are unknown components that are difficult to account for in the training data used to develop fully automated techniques.

The Supplementary Table provides a non-exhaustive list of commercially available liver segmentation software and solutions that can be used for volumetry and 3-D modelling. These software solutions employ various semi-automated and automated segmentation techniques in combination with the ability to perform manual corrections. For each software solution, we have provided the following information: manufacturer, operating system supported, segmentation techniques employed, modalities supported, sequences or vascular phases recommended, ability to perform subsegmentation, PACS integration, and a web page reference.

In addition, liver segmentation and surgical planning services are offered by private companies according to a fee-for-service model. Anonymised datasets are uploaded to a website and segmentation results are returned with 3-D models that may be viewed on a website, in a dynamic document, or within a dedicated software viewer for simulation of surgical scenarios. An example can be found in the Supplementary Table.

### MRI-based biomarkers

Imaging-based biomarkers have recently been introduced for quantification of diffuse liver disease. MRI-determined proton density fat fraction (PDFF) [62–64] has become an alternative to liver biopsy for estimation of liver fat content [62] and produces parametric maps of fat distribution throughout the liver [65]. The product of the average PDFF and the segmented liver volume produces the total liver fat index (TLFI), a novel biomarker of total fat burden in non-alcoholic steatohepatitis. TLFI has been shown to accurately monitor liver fat burden over time in the setting of a clinical trial [66]. Other volume averaged biomarkers in liver disease, such as iron per unit volume, are also being investigated. Further studies may incorporate other volume averaged biomarkers in liver disease [67].

## Impact of liver quality

While the bulk of this review has focused on the determination of liver volume—which directly impacts procedural planning—liver quality is an important, and interrelated, parameter. As alluded to in the section “Indications for liver volumetry”, the optimal ratio of FLR to TLV varies depending on the degree of liver pathology; higher FLR to TLV ratios are required in those with moderately diseased livers (i.e. liver steatosis secondary to chemotherapy, obesity or type 2 diabetes mellitus) and cirrhosis compared to normal prior to hepatectomy [17, 68].

Liver quality has an impact on liver segmentation as well. For example, fatty liver secondary to chemotherapy appears less dense, which may make hypovascular liver metastases more difficult to detect [69]. In the setting of cirrhosis, animal studies have demonstrated reduced uptake of gadoxetate disodium related to reduced expression of organic anion-transporting polypeptides [69, 70]. Clinically, an increase in hepatobiliary phase enhancement ratios has been observed in patients with Child-Pugh A disease compared to Child-Pugh C disease [71]. Thus, the benefit of liver uptake of hepatobiliary contrast agents in segmentation may be decreased with advancing liver disease. Increasing the dose of contrast agent or increasing the flip angle in these patients may help compensate for lower liver signal [72].

## Conclusions

Liver volumetry is a common application of liver segmentation. Liver volumetry is indicated in procedural planning for hepatectomy, PVE, ALPPS and liver transplant. Liver segmentation can be performed manually or with the assistance of semi-automated or fully automated algorithms of both CT and MR images. Future studies will be directed at addressing segmentation pitfalls, incorporating patient-specific subsegmentation and 3-D modelling into clinical decision making, the use of novel contrast agents and the combination of liver volumetry with MRI-based biomarkers.

## Electronic supplementary material

ESM 1 (DOCX 38 kb)

ESM 2 (MOV 793 kb)

ESM 3 (MOV 1282 kb)

ESM 4 (MOV 1330 kb)

ESM 5 (MOV 9515 kb)

ESM 6 (MOV 727 kb)
