# Supplementary material for: Liver segmentation: indications, techniques and future directions
Source: Insights Imaging. 2017 Jun 14;8(4):377–92. doi: 10.1007/s13244-017-0558-1 (PMC5519497; doi:10.1007/s13244-017-0558-1)
Supplement: Supplementary file 1 — (DOCX 38 kb) [file 13244_2017_558_MOESM1_ESM.docx]

**Supplementary Table**. List of commercially available liver segmentation software and solutions

| Software | Manufacturer | Operating System | Segmentation techniques employed | Modality supported | Sequence or vascular phase recommended | Liver subsegmentation | PACS Integration | Webpage |
| --- | --- | --- | --- | --- | --- | --- | --- | --- |
| *syngo*.CT Liver Analysis | Siemens Healthcare GmbH | Windows | Manual, active contours, livewire, shape interpolation, 3D deformable models, nudge | CT and MRI (view only) | CT:  Liver: Portal venous  Lesion: Depends on lesion  Vessel: Density difference of at least 20 HU between parenchyma and vessels. Same series applied to both hepatic and portal veins for optimal results.  MRI:  Cholangiograms for the bile ducts | Liver  Lesion  Vessel  Bile duct | No | https://www.healthcare.siemens.com/  computed-tomography/clinical-imaging  -solutions/ct-oncology-engine |
| Myrian | Intrasense | Windows. Thin client version for Windows server. | Fully automated. Data-driven hierarchical region growing. Edge properties used as well. | CT.  MRI in development. | Portal venous phase is preferred. Arterial, late phase, and unenhanced also acceptable. | Lesion  Vascular | Yes, many options available. | http://www.intrasense.fr/myrian/ |
| Hepatic VCAR | GE | Linux and Windows | Probabilistic model based iterative segmentation methods | CT | All phases can theoretically be used. Portal venous phase is typically optimal. | Liver  Lesion  Vessel | Yes, with all PACS that can interface with AW Server. | http://www3.gehealthcare.com/en/  products/categories/advanced_  visualization/applications/hepatic_vcar |
| LD2. Lobular Decomposition Tool | Terarecon | Windows | Automatic active contours, shape interpolation, region growing, manual ROI | CT | Multiple phases (e.g. arterial and venous) | Lesion  Vascular | Yes: All major PACS vendors | http://www.terarecon.com/advanced-visualization/liver-segmentation-package |
| MeVisDistant Services | Provided by MeVis | N/A: Results provided as classic PDF, interactive PDF, and video. | Performed by radiology technicians. Includes semi-automatic (livewire) and manual techniques (delineation, in-painting, masking). | CT and MRI.  Fusion of segmentation results from CT and MRI (especially MRCP) is possible. | CT:  Liver and venous anatomy: Venous and optional late venous phase  Artery: Arterial phase  MRI:  Cholangiograms for the bile ducts | Liver  Lesion  Vessel  Bile duct | N/A. However, results can be viewed in PACS. | http://www.mevis.de/loesungen/  professional/mevis-distant-services-mds |

**Supplementary Material 1 -** Manual Segmentation

**Supplementary Material 2 -** Active Contours

**Supplementary Material 3** - Livewire

**Supplementary Material 4** - Region Growing

**Supplementary Material 5** - Graph Cut
